# WHO STRESSES URGENT NEED FOR R&D FOR DRUG-RESISTANT TB ALONGSIDE NEWLY-PRIORITIZED ANTIBIOTIC-RESISTANT PATHOGENS

**Published:** 2017-04

**28 FEBRUARY 2017 | GENEVA** - WHO reaffirms the critical need for research and development (R&D) of new antibiotics to tackle the threat of drug-resistant tuberculosis (TB).

“Addressing drug-resistant TB research is a top priority for WHO and for the world,” said Dr Margaret Chan, WHO Director-General. “More than US$ 800 million per year is currently necessary to fund badly needed research into new antibiotics to treat TB.”

The MDR-TB public health crisis continues: there were an estimated 580 000 cases and 250 000 related deaths in 2015. Only 125 000 were started on treatment, and just half of those people were cured.

Only two new antibiotics to address MDR-TB have completed Phase IIB trials in the past 50 years. Both are still in Phase III trials, and more funding will be required to complete the process and to develop other effective treatment regimens.

On 27 February, WHO published a list of antibiotic-resistant pathogens that have recently been prioritized as posing great risk to human health.

“Mycobacterium tuberculosis, the bacterium responsible for human TB, was not included in the scope of the prioritization exercise as the intention was to identify previously unrecognised health threats due to increasing antibiotic resistance. There is already consensus that TB is a top priority for R&D for new antibiotics,” said Dr Marie-Paule Kieny, Assistant Director-General at WHO.

A series of high-level global meetings on TB have been scheduled in 2017-2018. Drug-resistant TB and research will be major themes at the WHO Ministerial Conference on TB planned in Moscow in November 2017. It will also be a key agenda item at the UN General Assembly high-level meeting on TB in 2018. MDR-TB and research needs are also under discussion in wider fora such as those focusing on antimicrobial resistance and health security.

Available from: http://www.who.int/mediacentre/news/releases/2017/drug-resistant-tb/en/

